# *Salmonella* Genomic Island 1B Variant Found in a Sequence Type 117 Avian Pathogenic Escherichia coli Isolate

**DOI:** 10.1128/mSphere.00169-19

**Published:** 2019-05-22

**Authors:** Max Laurence Cummins, Piklu Roy Chowdhury, Marc Serge Marenda, Glenn Francis Browning, Steven Philip Djordjevic

**Affiliations:** a The ithree institute, University of Technology Sydney, Ultimo, New South Wales, Australia; b Asia-Pacific Centre for Animal Health, Department of Veterinary Biosciences, Faculty of Veterinary and Agricultural Sciences, The University of Melbourne, Parkville, Victoria, Australia; c Asia-Pacific Centre for Animal Health, Department of Veterinary Biosciences, Faculty of Veterinary and Agricultural Sciences, The University of Melbourne, Werribee, Victoria, Australia; Escola Paulista de Medicina/Universidade Federal de São Paulo

**Keywords:** *Escherichia coli*, One Health, *Salmonella* genomic island 1, antibiotic resistance, avian pathogenic *E. coli*, genomics, multidrug resistance, poultry, veterinary microbiology, veterinary pathogens, whole-genome sequencing, zoonotic infections

## Abstract

*Salmonella* genomic island 1 (SGI1) is an integrative genetic island first described in Salmonella enterica serovars Typhimurium DT104 and Agona in 2000. Variants of it have since been described in multiple serovars of S. enterica, as well as in Proteus mirabilis, Acinetobacter baumannii, Morganella morganii, and several other genera. The island typically confers resistance to older, first-generation antimicrobials; however, some variants carry *bla*_NDM-1_, *bla*_VEB-6_, and *bla*_CTX-M15_ genes that encode resistance to frontline, clinically important antibiotics, including third-generation cephalosporins. Genome sequencing studies of avian pathogenic Escherichia coli (APEC) identified a sequence type 117 (ST117) isolate (AVC96) with genetic features found in SGI1. The complete genome sequence of AVC96 was assembled from a combination of Illumina and single-molecule real-time (SMRT) sequence data. Analysis of the AVC96 chromosome identified a variant of SGI1-B located 18 bp from the 3′ end of *trmE*, also known as the *attB* site, a known hot spot for the integration of genomic islands. This is the first report of SGI1 in wild-type E. coli. The variant, here named SGI1-B-Ec1, was otherwise unremarkable, apart from the identification of IS*Ec43* in open reading frame (ORF) S023.

**IMPORTANCE** SGI1 and variants of it carry a variety of antimicrobial resistance genes, including those conferring resistance to extended-spectrum β-lactams and carbapenems, and have been found in diverse S. enterica serovars, Acinetobacter baumannii, and other members of the Enterobacteriaceae. SGI1 integrates into Gram-negative pathogenic bacteria by targeting a conserved site 18 bp from the 3′ end of *trmE*. For the first time, we describe a novel variant of SGI1 in an avian pathogenic Escherichia coli isolate. The presence of SGI1 in E. coli is significant because it represents yet another lateral gene transfer mechanism to enhancing the capacity of E. coli to acquire and propagate antimicrobial resistance and putative virulence genes. This finding underscores the importance of whole-genome sequencing (WGS) to microbial genomic epidemiology, particularly within a One Health context. Further studies are needed to determine how widespread SGI1 and variants of it may be in Australia.

## Abstract (precis)

SGI1 and variants of it carry a variety of antimicrobial resistance genes, including those conferring resistance to extended-spectrum β-lactams and carbapenems, and have been found in diverse S. enterica serovars, Acinetobacter baumannii, and other members of the Enterobacteriaceae. SGI1 integrates into Gram-negative pathogenic bacteria by targeting a conserved site 18 bp from the 3′ end of *trmE*. For the first time, we describe a novel variant of SGI1 in an avian pathogenic Escherichia coli isolate. The presence of SGI1 in E. coli is significant because it represents yet another lateral gene transfer mechanism to enhancing the capacity of E. coli to acquire and propagate antimicrobial resistance and putative virulence genes. This finding underscores the importance of whole-genome sequencing (WGS) to microbial genomic epidemiology, particularly within a One Health context. Further studies are needed to determine how widespread SGI1 and variants of it may be in Australia.

## OBSERVATION

*Salmonella* genomic island 1 (SGI1) is a site-specific, integrative genetic element that uses a tyrosine recombinase encoded by *int*_SGI1_ to target the terminal 18 nucleotides (*attB*) of the *trmE* (formerly *thdF*) gene, which encodes a highly conserved GTPase (1). A toxin-antitoxin system (*sigAT*) encoded within SGI1 plays a critical role in its stable maintenance in the host chromosome (2), and while the island can excise as a circularized form via a process that requires *int*_SGI1_ (1), the frequency at which this occurs in the wild is thought to be very low and is not well understood (3). The transcriptional regulator complex AcaCD encoded by genes on IncA/C plasmids is sufficient to trigger excision and mobilization of SGI1 (1, 4), yet IncA/C plasmids are not known to coexist in the same host as SGI1, suggesting that an active exclusion mechanism limits opportunities for transposition. These observations in part explain why SGI1 is stably maintained in the chromosome, the difficulties encountered in assaying for circular forms of SGI1 (low abundance), and the apparent low transposition frequency of the island (1, 5).

SGI1 comprises a backbone of 27.4 kb and a complex class 1 integron (In104) of 15 kb that resides in *resG* (open reading frame [ORF] S027). In104 is flanked by a 5-bp duplication consistent with transposition into *resG.* Variations in the size of In104 arise depending on the resistance gene cargo it carries, homologous recombination events between shared sequences within the integron, the presence of other mobile elements, and the action of IS elements (6), particularly IS*26*. The introduction of IS*26* in SGI1 creates further opportunities for the acquisition of diverse antibiotic resistance genes and the rapid evolution of these elements. Notable in this regard is SGI1-L2, which carries an IS*26*-flanked composite transposon containing multiple antibiotic resistance genes in S024 (7). IS elements such as IS*Vch4* (IS*1359*) are associated with deletions in the SGI1 backbone, and these events contribute to the ongoing evolution of the element.

SGI1 and variants of it may be able to integrate into a wide variety of Gram-negative bacteria because the sequence of the terminal 18 nucleotides of *trmE* (*attB*) is well conserved (8). Experiments performed *in vitro* have demonstrated that SGI1 is able to integrate into Klebsiella pneumoniae and Escherichia coli (9), but evidence of the presence of the island in these species in natural environments has been lacking. Since the identification of SGI1 in Salmonella enterica serovar Typhimurium DT104 almost 20 years ago, homologous recombination events, as well as insertion sequence-mediated indels, have led to the emergence of more than 30 SGI1 variants, some of which carry antimicrobial resistance genes that are of major clinical significance (10). SGI1 and variants of it have been detected in diverse serovars of S. enterica and other Gram-negative pathogens (6, 11–14). For example, *Proteus* genomic island 1 (PGI1), identified in Proteus mirabilis, carries extended-spectrum β-lactamase and/or metallo-β-lactamases (15, 16), and SGI variants have been reported in Morganella morganii subsp. *morganii* (10), Acinetobacter baumannii (17), Enterobacter hormaechei subsp. *oharae* (18), and Providencia stuartii (19).

While performing an *in silico* analysis of whole-genome sequencing (WGS) data from 97 Australian avian pathogenic E. coli (APEC) isolates (20), one isolate (AVC96) from a diseased 26-week-old broiler chicken was found to carry genetic signatures typically found in SGI1 (GenBank accession no. AF261825). Details of the materials and methods used for analysis of the isolate are given in Text S1 in the supplemental material. Sequence analysis identified AVC96 as an APEC isolate with sequence type 117 (ST117), a lineage associated with extraintestinal infections in humans and poultry (21). A hybrid assembly using the program Unicycler, which combined Illumina short reads and single-molecule real-time (SMRT) sequences derived from a Pacific Biosystems RSII sequencer, resolved the structure of the SGI1 variant in isolate AVC96 and placed it a single 4,886,273-bp chromosomal contig. The SGI1 variant was inserted in the terminal 18 bp of *trmE*. The variant of SGI1 was here named SGI1-B-Ec1.

10.1128/mSphere.00169-19.1 TEXT S1 Supplemental materials and methods. Download Text S1, DOCX file, 0.03 MB. Copyright © 2019 Cummins et al. 2019 Cummins et al. This content is distributed under the terms of the Creative Commons Attribution 4.0 International license.

**FIG 1:**
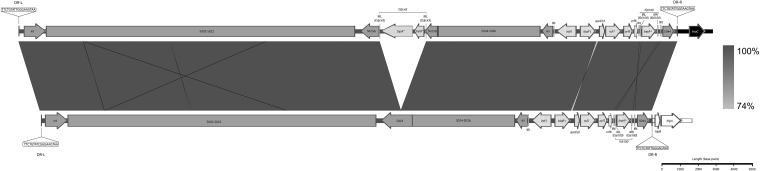
Schematic showing the structural homology between SGI1-B-Ec1 (top) and SGI1-B (bottom). Left and right direct repeats (DR-L and DR-R, respectively) are shown flanking either element. Integron-associated elements are shown with a crosshatched pattern, while other elements of the SGI-1 backbone are shown in dark gray. Genetic elements downstream of SGI1-B-Ec1 are shown in black, while those downstream of SGI1-B are shown in white. ORFs and inverted repeats of IS element IS*Ec43*, unique to SGI1-B, are shown with a dotted pattern near the center of the element. “tnpA*” and “tnpB*” are IS*Ec43* associated, and “*tnpA*^” is IS*6100* associated. Note that genomic coordinates are not to scale and are only approximate. See the GenBank entry for SGI1-B-Ec1 (accession no. MK599281) for precise feature coordinates.

Comparative analysis with published SGI1 reference sequences revealed that the structure of SGI1-B-Ec1 in isolate AVC96 is related to SGI-1B (accession no. KU987430), as seen in Fig. 1. A homologous recombination event between the copies of *intI1* resulted in the loss of the intervening DNA, a feature of this variant. SGI1-B-Ec1 differs from SGI1-B and other SGI1 variants via the insertion of IS*Ec43* in S023. IS*Ec43* is flanked by an 8-bp direct repeat, suggesting its integration is a recent event. The location of IS*Ec43* in S023 has not been previously described, and it may serve as a unique epidemiological marker for tracking isolates that carry SGI1-B-Ec1 in Australia. SGI1-B-Ec1 also carries a unique single nucleotide polymorphism within *qacE*Δ*1* (228 bp).

In E. coli, *trmE* sits proximal to *tnaC*, which encodes a tryptophanase. In the case of AVC96, SGI1-B-Ec1 sits between these ORFs. An analysis of 455,632 bacterial whole-genome sequence data sets in the short-read archive (22) indicated that none of the approximately 38,000 E. coli genomes available therein carry an SGI1 variant at this locus. BLASTn analysis of the publicly available nucleotide database yielded one entry (GenBank accession no. KU842063.1) that spanned from base 31 of S044 to base 153 of *tnaA.* This sequence was the derived from an *in vitro* experiment that sought to determine the ability of SGI1 to integrate into E. coli (9). Therefore, our findings support the contention that AVC96 is the first description of the occurrence of a variant of SGI1 in wild-type E. coli. It is notable that variants of SGI1 carrying *bla*_NDM-1_ (23), *bla*_VEB-6_ and *qnrA1* (15), and *bla*_CTX-M-15_ (24) have been identified in multiple drug-resistant Proteus mirabilis and Salmonella enterica isolates. This discovery should prompt investigations on the prevalence of SGI1-B-Ec1 in Australia and how it might evolve to capture a broader selection of antimicrobial resistance genes.

## 

### Data availability.

Long-read whole-genome sequence data and short-read whole-genome sequence data are available in the SRA under accession no. SRR8671292 and SRR7469869, respectively, while the nucleotide sequence of SGI1-B-Ec1 is available on the NCBI nucleotide database under accession no. MK599281.
