# Supplementary material for: Salmonella Genomic Island 1B Variant Found in a Sequence Type 117 Avian Pathogenic Escherichia coli Isolate
Source: mSphere. 2019 May 22;4(3):e00169-19. doi: 10.1128/mSphere.00169-19 (PMC6531882; doi:10.1128/mSphere.00169-19)
Supplement: TEXT S1 [file mSphere.00169-19-s0001.docx]

*Salmonella* Genomic Island 1B Variant Found in a Sequence Type 117 Avian Pathogenic *Escherichia coli* Isolate

Max Laurence Cummins^1^, Piklu Roy Chowdhury^1^, Marc Serge Marenda^2^, Glenn Francis Browning^2^ and Steven Philip Djordjevic^1^*

1 The ithree Institute, University of Technology Sydney, Ultimo, NSW, Australia,

2 Asia-Pacific Centre for Animal Health, Department of Veterinary Biosciences, Faculty of Veterinary and Agricultural Sciences, The University of Melbourne, Parkville, Victoria 3010, and Werribee, Victoria 3030, Australia.

*Corresponding author

Steven P. Djordjevic

Email: steven.djordjevic@uts.edu.au

Supplemental Materials and Methods Section

Sample collection

AVC96 was collected in 2009 from a 26-week old diseased commercial chicken that suffered from a presumptive Avian pathogenic *E. coli* infection. The sample was cultured on sheep blood and MacConkey agar as well as subjected to a routine PCR (1) to enable its identification as putative APEC. It was subsequently store at -80°C in 20% glycerol or on Protect (Thermo-Fisher) beads. __________________________________________________________________________________

DNA isolation and Illumina short-read sequencing

After overnight culturing in 5 mL of Lysogeny broth (LB), genomic DNA was extracted using the ISOLATE II Genomic DNA Kit (Bioline) following the manufacturer’s instructions and stored at -20°C. Nextera® DNA Library Preparation kits were used to generate 150-bp paired end reads from 0.5ng of template DNA before whole genome sequencing using an Illumina HiSeq® 2500. FastQC version 0.11.5 was used to quality assess read quality. __________________________________________________________________________________

High molecular weight DNA isolation for long read sequencing

Genomic DNA for use long read sequencing was isolated using a custom protocol combining the protocols of Tillett and Neilan (2) and the QIAGEN© Blood and cell culture DNA Midi kit.

Firstly, LB was inoculated and cultured overnight. A 6 mL aliquot was then pelleted at 8000rpm for 6 mins before cells were resuspended in 50 uL TER (TE buffer with 200ug/mL RNAse A). 1 mL of xanthogenate-SDS buffer (2) was added to the resuspended cells which were incubated at 50°C for 2 hours or until complete lysis was observed.

Tubes were vortexed for 10 seconds before being put on ice for 30 minutes and spun at 4°C at 14000rpm for 10 minutes before the supernatant was transferred to a new tube. The DNA was then precipitated with a 1:1 ratio of room temperature isopropanol with gentle agitation. Tubes were then spun at 4°C at 14000rpm for 10 minutes before pouring off of isopropanol, pellets were washed in 1 mL of 70% ethanol and the centrifugation was repeated. Ethanol was then poured off and the tubes were allowed to air dry in a laminar flow hood. The pellet was then resuspended in 500uL buffer B1 from the QIAGEN© kit and left at 50°C overnight.

The next morning tube contents (including any remaining pellet) were transferred to a 15 mL falcon tube and an additional 3 mL of buffer B1 was added. The protocol was subsequently carried out from step five in the Qiagen Genomic Manual, however note that here QIAGEN© Proteinase K was added but RNAse A, typically required by the protocol, was not included as they were added during the lysis step. __________________________________________________________________________________

Single Molecule Real-Time (SMRT) cell long-read sequencing

Long read sequencing was undertaken at the Ramaciotti Centre for Genomics using a Pacific Biosciences RSII sequencer with P6-C4 chemistry on a SMRT Cell.

__________________________________________________________________________________

Long-read sequence pre-processing and assembly

Long reads were filtered using filtlong with the following command:

filtlong -1 AVC96_R1.fastq.gz -2 AVC96_R2.fastq.gz --min_length 1000 --keep_percent 90 --target_bases 500000000 A96_NovemberPacBio.fastq.gz | gzip > AVC96_filtered.fastq.gz

Subsequently, filtered short reads and long reads were combined in a hybrid assembly using Unicycler as follows:

unicycler -1 AVC96_R1.fastq.gz -2 AVC96_R2.fastq.gz -l AVC96_filtered.fastq.gz -o AVC96_hybrid

__________________________________________________________________________________

EasyFig

The coordinates of the SGI1-B-EC1 structure from the hybrid assembly of AVC96 sequence reads were identified using ISfinder (<https://isfinder.biotoul.fr)> and Megablast (<https://blast.ncbi.nlm.nih.gov/Blast.cgi)> with SGI1B (Accession No.:KU987430) as a reference sequence. The 35,728 kb sequence of SGI1-B-EC1 (bases 4,299,832-4,335,559) was extracted from a 4,886,273 bp long scaffold from the assembly saved as a separate fasta file using Snapgene ([www.snapgene.com)](http://www.snapgene.com)).

This resulting fasta file was fed into Easyfig to produce a bitmap image of the homology between SGI1-B-EC1 and SGI1B:

Easyfig.py -o figure.bmp SGI1-B-EC1.fasta SGI1B.fasta

Read-mapping

Short reads were mapped to the SGI1-B-EC1 sequence produced above to check for read depth across the element with Burrows-Wheeler Aligner using the following commands:

bwa mem -t16 -MY SGI1-B-EC1.fasta AVC96_R1.fastq.gz AVC96_R2.fastq.gz | samtools view -ubS -F 0x904 - | samtools sort -@8 -T SGI1-B-EC1.fasta - -o AVC96_SGI1-B-EC1.bam

samtools depth AVC96_SGI1-B-EC1.bam > AVC96_SGI1-B-EC1_short-read_depth.txt

__________________________________________________________________________________

To facilitate visualization of the read alignment, the following command was used before loading the bam and associated reference sequence in Tablet to confirm the bridging of the SGI1-B-EC1 structure to the chromosomal context by sequencing reads.

samtools index AVC96_SGI1-B-EC1.bam

__________________________________________________________________________________

Long reads were mapped to the same SGI1-B-EC1 sequence using minimap2 to check for the depth across the structure. This was done using the following commands:

minimap2 -ax map-pb SGI1-B-EC1.fa AVC96_filtered.fastq.gz > SGI1-B-EC1.sam

samtools view -hF 0x904 SGI1-B-EC1.sam | samtools sort -@8 -T SGI1-B-EC1.fa - -o SGI1-B-EC1.sam

samtools depth SGI1-B-EC1.sam > SGI1-B-EC1_long-read_depth.txt

__________________________________________________________________________________

Again, Tablet was used to confirm that PacBio reads bridged the junctions between the SGI1-B-EC1 element and the chromosome of AVC96, however due to a difference in minimap2 output format the alignment file had to be indexed using tabix after zipping the file with bgzip.

bgzip SGI1-B-EC1.sam

tabix -p sam SGI1-B-EC1.sam.gz

__________________________________________________________________________________

Snapgene and SGI1-B-EC1 schematic annotation

Snapgene annotations for SGI1B were created using a GenBank file as a template before custom colouring and labelling of ORFs.

Snapgene annotations for SGI1-B-EC1 were created through the importation of annotated features from a GenBank file of SGI1B before subsequent confirmation using Megablast with SGI1B (Accession No.: KU987430) as a reference sequence. ISEc43, the insertion sequence inserted into S023, was identified using ISfinder (<https://isfinder.biotoul.fr/)>.

These annotations were used to make schematics of SGI1-B-EC1 and SGI1-B which were added atop and below the EasyFig bitmap image, respectively, using image editing software.

__________________________________________________________________________________

Program sources and versions:

| **Program** | **Version** | **URL** | **Manuscript**  **Reference** |
| --- | --- | --- | --- |
| Minimap2 | 2.12-r827 | <https://github.com/lh3/minimap2> | (3) |
| BWA | 0.7.17-r1188 | <https://github.com/lh3/bwa> | (4) |
| Samtools | 1.9 | <http://samtools.sourceforge.net/> | (5) |
| EasyFig | 2.2.3 | <http://mjsull.github.io/Easyfig/> | (6) |
| Tablet | 1.17.08.17 | <https://ics.hutton.ac.uk/tablet/> | (7) |
| Unicycler | V0.3.0b | <https://github.com/rrwick/unicycler> | (8) |
| Filtlong | v0.2.0 | <https://github.com/rrwick/Filtlong> | N/A |
| Snapgene | 4.1.9 | <http://www.snapgene.com/> | N/A |

__________________________________________________________________________________

References

1. Johnson TJ, Wannemuehler Y, Doetkott C, Johnson SJ, Rosenberger SC, Nolan LK. 2008. Identification of minimal predictors of avian pathogenic Escherichia coli virulence for use as a rapid diagnostic tool. J Clin Microbiol 46:3987-96.

2. Tillett D, Neilan BA. 2000. Xanthogenate nucleic acid isolation from cultured and environmental cyanobacteria. Journal of Phycology 36:251-258.

3. Li H. 2018. Minimap2: pairwise alignment for nucleotide sequences. Bioinformatics 1:7.

4. Li H, Durbin R. 2009. Fast and accurate short read alignment with Burrows–Wheeler transform. Bioinformatics 25:1754-1760.

5. Li H, Handsaker B, Wysoker A, Fennell T, Ruan J, Homer N, Marth G, Abecasis G, Durbin R. 2009. The sequence alignment/map format and SAMtools. Bioinformatics 25:2078-2079.

6. Sullivan MJ, Petty NK, Beatson SA. 2011. Easyfig: a genome comparison visualizer. Bioinformatics 27:1009-1010.

7. Milne I, Bayer M, Cardle L, Shaw P, Stephen G, Wright F, Marshall D. 2009. Tablet—next generation sequence assembly visualization. Bioinformatics 26:401-402.

8. Wick RR, Judd LM, Gorrie CL, Holt KE. 2017. Unicycler: resolving bacterial genome assemblies from short and long sequencing reads. PLoS computational biology 13:e1005595.
